# Spanish transcultural adaptation of the 4AT score for the evaluation of delirium in the emergency department: a prospective diagnostic test accuracy study

**DOI:** 10.1186/s12912-023-01638-6

**Published:** 2024-02-06

**Authors:** Marta Morales-Puerto, María Ruiz-Díaz, Silvia García-Mayor, Álvaro León-Campos, José Miguel Morales-Asencio, José Carlos Canca-Sánchez, Sonia Gavira-Guerra, Cecilia Toledo-Fernandez, Marta Aranda-Gallardo

**Affiliations:** 1 Hospital Costa del Sol. Servicio Andaluz de Salud, Autovía A7, Km. 187. Marbella, Malaga, 29603 Spain; 2 Faculty of Health Sciences, Department of Nursing, Universidad de Málaga, C/ Arquitecto Francisco Peñalosa 3, Malaga, 29017 Spain; 3 Instituto de Investigación Biomédica de Málaga (IBIMA-Bionand), C/ Miguel Díaz Recio, Malaga, 29010 Spain; 4 Red de Investigación en Cronicidad, Atención Primaria y Prevención y Promoción de la Salud (RICAPPS), Instituto de Salud Carlos III, Bizkaia, Spain

**Keywords:** Delirium, 4AT, Screening tool, Adverse events, Multimorbidity, Validation study, Nursing

## Abstract

**Background:**

Delirium is one of the most common adverse events in older people during hospitalization, especially in the emergency department. Reliable, easy-to-use instruments are necessary to properly manage delirium in this setting. This study aims to evaluate the diagnostic validity of the Spanish version of the 4 ‘A’s Test (4AT) in the ED.

**Methods:**

A diagnostic accuracy study was conducted in patients over 65 years old admitted to the Emergency Department who did not have a formal diagnosis of dementia or a severe mental health disorder. Face and content validity were evaluated by an expert panel. Emergency nurses performed the evaluation with 4AT, whilst blinded and trained researchers assessed patients with the Revised Delirium Rating Scale as the gold standard. The content validity index, sensitivity, specificity, positive and negative predictive values, likelihood ratios, Youden’s Index and ROC curves were calculated to evaluate the diagnostic accuracy of the instrument.

**Results:**

Of 393 eligible patients, 380 were finally analyzed. Content validity yielded a median content validity index of 4 (interquartile range: 0). The Spanish 4AT sensitivity (95.83%; 95% ECI: 78.9–99.9%), specificity (92.98%; 95% CI: 89.8–95.4%), positive predictive value (47.92%) and negative predictive value (99.7%) were satisfactory. Youden’s index was 0.89. Positive likelihood ratio was 13.65, and negative likelihood ratio 0.045. The area under the curve was 0.97.

**Conclusions:**

The Spanish version of the 4AT for use in the Emergency Departments is easy-to-use and applicable. The validation results indicate that it is a valid instrument with sufficient predictive validity to identify patients at risk of delirium in the Emergency Departments. Moreover, it is a tool that facilitates the management of an adverse event that is associated with increased mortality and morbidity.

## Introduction

### Background

According to the Diagnostic and Statistical Manual of Mental Disorders (DSM-5TM) criteria, delirium implies a disturbance in attention and awareness, over a short period of time, represents a change from baseline attention and awareness, and tends to fluctuate in severity during the course of a day. These changes are not better explained by a pre-existing, established or evolving neurocognitive disorder and do not occur in the context of a severely reduced level of arousal. It may be accompanied by disturbance in cognition. Moreover, there is evidence from the history, physical examination or laboratory findings that the disturbance is a direct physiological consequence of another medical condition, substance intoxication or withdrawal, or exposure to a toxin, or is due to multiple etiologies [1].

The etiology of this syndrome is multifactorial, with predisposing factors such as age, multimorbidity or cognitive impairment, and precipitating factors such as drugs, severe infections or metabolic imbalance [2]. It has been associated with the use and discontinuation of drugs, medical and surgical processes, or the synergy of both factors [3]. Few cases are neurobiological in origin, although some precipitating factors, such as stroke, are recognized [4].

Delirium is one of the most common adverse events in hospitalized patients older than 65 years, with a prevalence of 23% (95% confidence interval [CI] 19-26%) [5]. It affects one in four hospitalized individuals [5], and one third of these cases can be prevented by multidisciplinary and non-pharmacological interventions [6]. In the emergency setting prevalence of delirium has been reported up 38% of older adults [7].

The state may persist for weeks or even months in 20% of cases [8], generating stress for both patients and their caregivers [9], prolonging their hospital stay [2], and increasing the risk of institutionalization, dementia and mortality by up to 8-fold [10, 11].

Emergency departments (EDs) are now prioritizing the prevention, detection and management of this adverse outcome [12].

The early, systematic detection of delirium is vitally important because it improves knowledge of its precipitating factors, helps establish treatment pathways according to severity, reduces the risk of falls, pain or other adverse events during hospitalization, and facilitates primary and secondary prevention [13].

More than five instruments are currently available for the evaluation of delirium in the ED, the most important of which are: the Confusion Assessment Method (CAM) [14], Modified Confusion Assessment Method for the Emergency Department (mCAM-ED) [15], Confusion Assessment Method for the Intensive Care Unit (CAM-ICU) [16], Delirium Triage Screen (DTS), Brief Confusion Assessment Method (bCAM) [17], Neelon and Champagne Confusion Scale (NEECHAM) [18], and the 4 ‘A’s Test (4AT) [19].

The 4AT was developed as a brief 4-item instrument to test alertness, orientation, attention, and fluctuating course [19]. It is currently one of the main tools recommended for the early detection of delirium [20], given its good diagnostic performance in the elderly population regardless of the clinical setting where it is applied [21].

However, no validation studies conducted in the EDs of Spanish hospitals have been identified. The aim of this study was to adapt the 4AT to the Spanish language and to evaluate its diagnostic accuracy for detecting delirium in the ED.

### Study objectives and hypotheses

#### General objective

To adapt the 4AT to the Spanish language and evaluate its validity for detecting delirium in patients admitted to the ED of the Hospital Costa del Sol, Marbella (Spain).

## Methods

### Design

This was a prospective diagnostic accuracy study conducted at the Hospital Costa del Sol (Spain). The study consisted of 2 phases: an initial cross-sectional cross-cultural adaptation and content validity phase, and a second diagnostic validation phase.

### Description of the translation process

The translation-back translation phase was carried out following the ISPOR guidelines [22] by a panel of experts, consisting of a native English-speaking scientific translator, and six bilingual nurses with more than five years of experience, whose mother tongue was Spanish. This phase began in December 2020 and was completed in February 2021.

Cross-cultural adaptation was divided into 4 stages:

1. **Translation of the original version**: It was carried out by two independent bilingual translators with 24 and 13 years of research experience, whose mother tongue was Spanish, to evaluate the divergent validity of the items (convergent terms, semantic discrepancies). The translated version was obtained, subsequently reviewed by the research team through a discussion panel, and harmonized until a joint version was reached.
2. **Back-translation**: A bilingual professional translator whose native language was English and blinded to the original version of the study, back-translated the version obtained in stage 1 in Spanish into the original language.
  Subsequently, semantic and conceptual discrepancies were analyzed by comparing them with the original scale in English.
3. **Semantic equivalence of the translation by a committee of experts**: The version that was translated into Spanish was submitted to review by a committee of experts of 15 care nurses in the ED department of the Costa del Sol Hospital, who had more than 5 years of experience caring for patients with delirium or at risk of it, evaluating the validity of aspect and content (clarity and understandability of each item) through an online Likert survey, with values ​​of 1 to 4 and 1 to 5 for relevance and comprehensibility respectively.

### Sample recruitment

The study population included patients older than 65 years who were admitted to the ED between March 2021 and December 2022 for a period of more than four hours and who did not show moderate cognitive impairment at the time of assessment (Pfeiffer questionnaire score < 6). The following participants were excluded: patients or family members who refused to sign informed consent or who subsequently requested withdrawal from the study; patients whose Pfeiffer score [23] was greater than 6 or whose medical history included a diagnosis of dementia or severe mental disorder according to the DSM-5 classification [1]; and patients transferred to the intensive care unit or to another hospital.

### Data collection

Sociodemographic and clinical characterization variables (Charlson index, reason for emergency care, diagnosis of dementia or cognitive impairment, hospital admission, and mortality) were collected. In addition to these variables, the 4AT was administered, along with the Revised Delirium Rating Scale (DRS-R-98) [24] that was used as the gold standard. The DRS-R-98 contains a total of 16 items: three for differential diagnosis and 13 for delirium severity. The severity section functions as a separate scale of repeated measures taken at short intervals during an episode of delirium; it can be administered by psychiatrists, psychologists, and nurses with prior training. The Spanish adaptation used in this study uses a cut-off point of 14 to establish the presence of delirium, without including the optional differential diagnosis items in the evaluation [25].

The 4AT score is a screening instrument designed for rapid and sensitive initial assessment of cognitive impairment and delirium in any healthcare setting. It consists of 4 items: (1) alertness, (2) AMT4 (abbreviated mental test), (3) attention, and (4) acute change or fluctuation in alertness or cognition. A score ≥ 4 suggests delirium, while a score between 1 and 3 suggests cognitive impairment. The validation studies carried out to date in the ED in other countries [26–28] maintain the same cut-off points as proposed by the original author.

To assess face and content validity, a panel of 15 clinical nurses with more than five years of experience in the ED used a Likert-type questionnaire with responses from 1 (totally disagree) to 5 (totally agree) to evaluate the relevance and comprehensibility of each of the items of the 4AT.

Parameters for the diagnostic validation phase were collected using an electronic system with data entry quality control. ED nurses administered the 4AT when patients were admitted to the ED, while the DRS-R-98 gold standard instrument was administered blindly by members of the research team trained in the use of this tool. These members also evaluated the Charlson score and the Pfeiffer test. The time between the administration of one scale and the other was never more than one hour.

### Statistical analysis

A total of 323 subjects were needed for a confidence level of 5% and to detect an expected sensitivity of 84% and specificity of 74%, assuming a 25% prevalence of delirium in the ED [26], with an accuracy of 8%. This sample size was increased by 20% to cover possible losses.

An exploratory analysis was carried out using measures of central tendency and dispersion, frequencies, and percentages. Normality of distribution of the variables was confirmed using the Kolmogorov-Smirnov test. To assess face and content validity, the median (Me) and interquartile range (IQR) of the responses provided by the experts were calculated. The degree of consensus was analyzed according to RAND corporation guidelines [29].

Diagnostic validity was assessed by calculating sensitivity, specificity, positive and negative predictive values, and positive and negative likelihood ratios (LR). The Youden index and the area under the ROC curve were also calculated. Convergent validity was also estimated with the Pfeiffer test and DRS 98 using correlation coefficients.

## Results

**Fig. 1:**
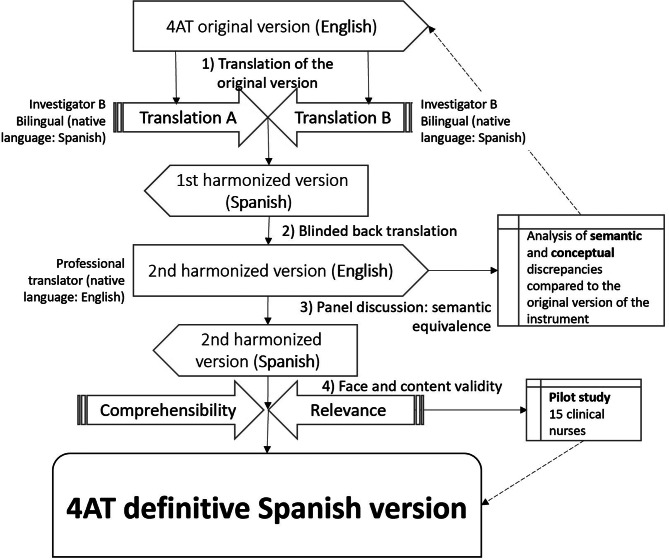
Translation and back translation for 4AT

For the analysis of comprehensibility and relevance of the items of both scales, the total sample of experts was 15. Their mean age was 33.4 years (standard deviation [SD]: 4.1), and they had an average of 3.4 years’ experience in the ED (SD: 1). Figure 1 shows the final process implemented for the adaptation to Spanish.

The 4AT required no major changes in its structure or content compared to the original instrument, and its semantic and cultural equivalence were both correct.

The level of relevance and comprehensibility of all the items in this scale was excellent (Table 1).

**Table 1:** 4AT face and content validity

			4AT n: 15				
**Relevance, content validity index (CVI) [Median (IQR)]**				**Comprehensibility [Median (IQR)]**			
*Item 1 Alertness*	*Item 2 Abbreviated mental test*	*Item 3 Attention*	*Item 4 Acute change or fluctuating course*	*Item 1: Alertness*	*Item 2: Abbreviated mental test*	*Item 3: Attention*	*Item 4: Acute change or fluctuating course*
4 (0)	4 (0)	4 (0)	4 (0)	5 (0)	5 (0)	5 (0)	5 (0)

**Fig. 2:**
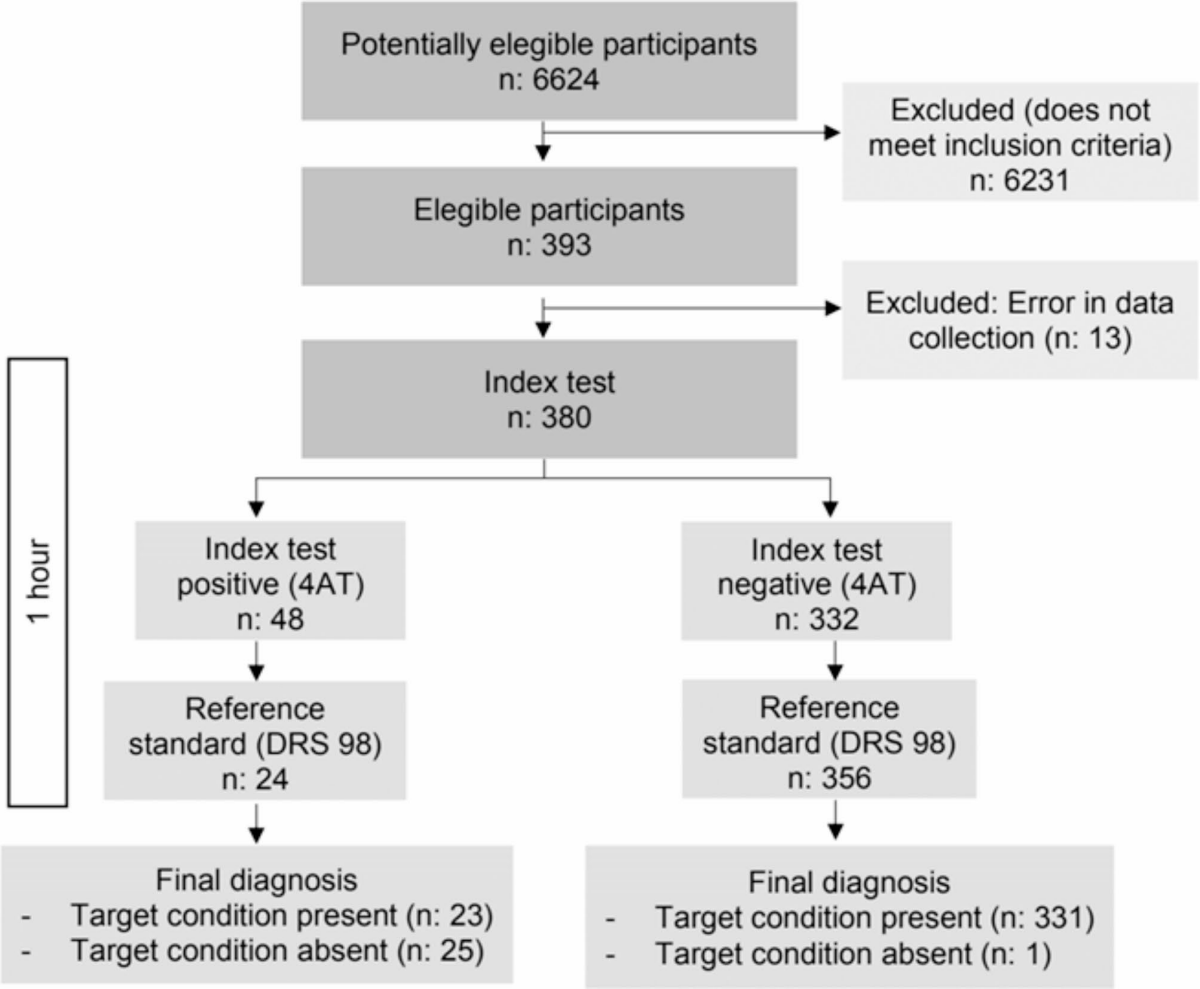
Study flowchart

The final sample consisted of 380 subjects with a mean age of 78.21 (SD: 7.38) years, (Median: 77; IQR: 11), 48.4% (n = 184) of whom were women. Figure 2 shows the study flow chart.

The mean Charlson index was 2.97 (SD: 2.13) points (Median: 2; IQR: 2). Just over half (52.9%) of the subjects were eventually admitted to the hospital. Of 380 subjects, 3.4% (n = 13) had mild cognitive impairment, with a mean Pfeiffer score of 1.81 (SD: 1.82) points (Median: 1; IQR: 3). The prevalence of delirium was 12.6% (48 subjects) using the 4AT, and 6.3% (24 subjects) using the DRS-R-98. There were no significant differences in the presence of delirium measured by either the 4AT or the DRS-R-98 among patients who were hospitalized compared to those who did not. The median age of subjects with delirium according to the 4AT was significantly higher (6.30 points; *p* < 0.001; Median: 83.50; IQR: 10). Furthermore, in patients with delirium, the risk of mortality after hospital discharge increased (*p* < 0.001). The level of cognitive impairment in subjects with delirium was significantly higher (*p* < 0.001) than in those without delirium.

At the selected cut-off point of ≥ 4, the 4AT detected 48 subjects with delirium (12.6%). Its sensitivity compared to the gold standard (DRS-R-98) was 95.83% (95% CI: 78.9-99.9%) with a specificity of 92.98% (95% CI: 89.8-95.4%), a positive predictive value (PPV) of 47.92%, and a negative predictive value (NPV) of 99.7%. The Youden index was 0.89. Table 2 provides a detailed breakdown of the 4AT’s accuracy in detecting delirium at various cut-off points, further illustrating its diagnostic performance.

**Table 2:** 4AT accuracy in detecting delirium according to the cut-off point

Cut-off point	Sensitivity (%)	Specificity (%)	PPV (%)	NPV (%)	Youden index
1	100	45.51	11.01	100	0.45
2	100	72.19	19.51	100	0.72
3	100	84.83	30.78	100	0.85
4	95.83	92.98	47.92	99.7	0.89
5	79.17	95.22	52.78	98.55	0.74
6	75	95.79	54.55	98.27	0.71
7	58.33	97.47	60.87	97.2	0.56
8	54.17	98.31	68.42	96.95	0.52

The positive LR (LR+) reached the value of 13.65, while the negative (LR-) was 0.045.

**Fig. 3:**
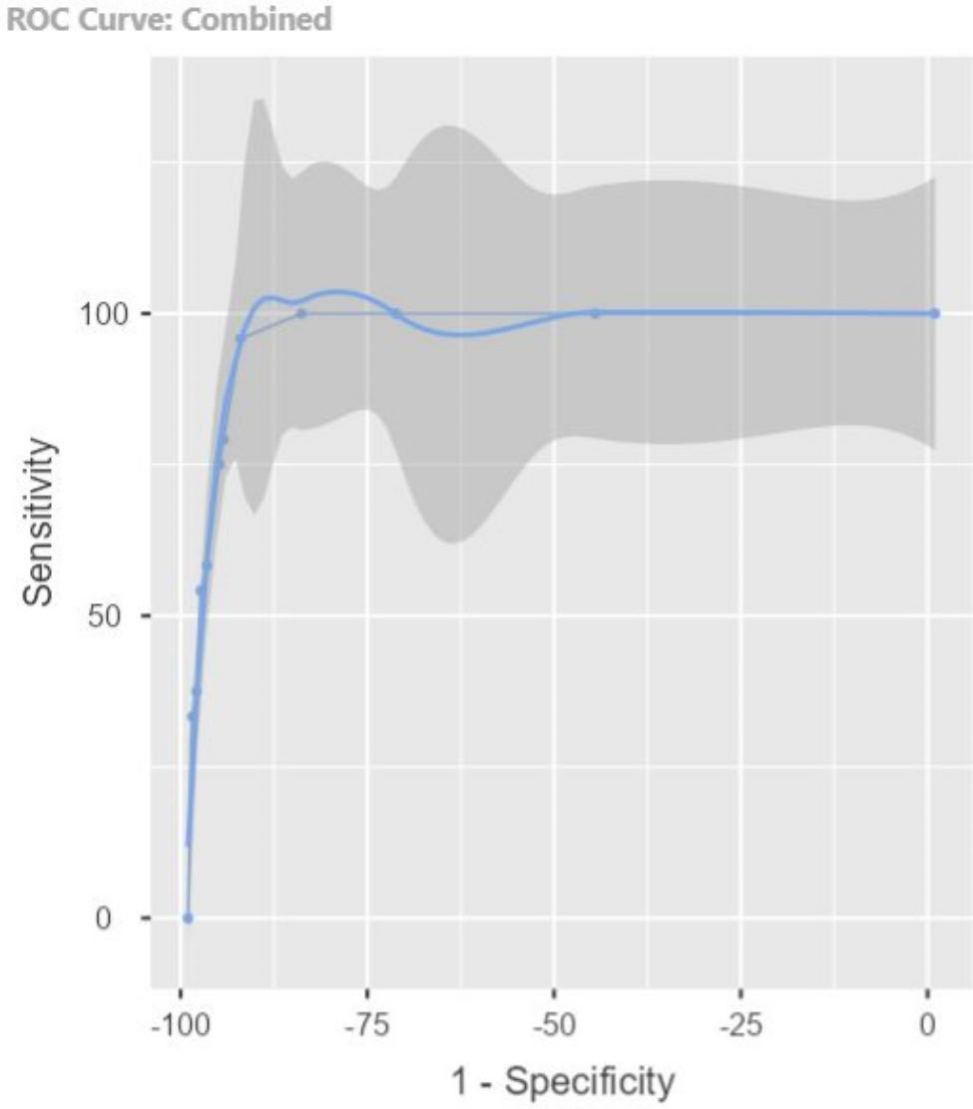
ROC curve for 4AT

Regarding the overall accuracy of the test, represented in Fig. 3, the area under the curve (AUC) was 0.97.

For the convergent validity of the instrument, a significant intraclass correlation coefficient of 0.62 (95% CI: 0.56–0.68)for the Pfeiffer test and 0.77 (95% CI: 0.73–0.81) for the DRS-R-98 were observed.

## Discussion

The aim of this study was to adapt the 4AT to Spanish for use in the ED, and to evaluate its diagnostic validity for delirium.

In terms of screening, in this study, the Spanish version of the 4AT score showed a good diagnostic performance in the ED, with a sensitivity of 95.83% (95% CI: 78.9-99.9%), specificity of 92.98% (95% CI: 89.8-95.4%), and an AUC of 0.974 for the cut-off point proposed by the original author [19].

The diagnostic accuracy of the 4AT has been established through meta-analysis, with an overall prevalence of delirium of 24.2% (95% CI [17.8–32.1%]), and a pooled sensitivity and specificity of 0.88 (95% CI [0.80–0.93]) and 0.88 (95% CI [0.82–0.92]) respectively [21].

The results of this study, both in terms of prevalence and diagnostic accuracy, seemed to be similar to another author´s investigations among older adults in the ED department. In one hand, Shenkin et al. [28] evinced a prevalence of 12.4% (0.2 points below from Spanish 4AT), while O`Sullivan et al. [27] and Gangé et al. [26] neither distanced more than 3 points (11% and 15,4% respectively),

On the other hand, sensitivity and specificity were also comparable. Shenkin et al. [28] reported 0.84 (95% CI: [0.76, 0.93]) for sensitivity and 0.74 (95% CI: [0.70, 0.78]) for specificity, while this study located sensitivity in 0.95 (95% CI [78.9–99.9%]) and specificity in 0.92 (95% CI [0.89–0.95]).

It isn´t different from the other two studies mentioned: O´Sullivan purposed a sensitivity of 0.93 (95% CI [0.83–0.98]), and a specificity of 0.91 (95% CI [0.88–0.94]) [28], and the French 4AT version from Gagné et al. [26] achieved a 0.84 sensitivity 95% CI: [0.76, 0.93]) and a 0.74 specificity (95% CI: [0.70, 0.78]).

Despite its high prevalence [30] and fatal outcomes [3, 31], delirium appears to be under-diagnosed in up to 60% of cases [32]. Research is still new and diverse [20, 33–35] and efforts need to focus on the screening, prevention and treatment of this entity and its consequences. The incidence of delirium in our study coincides with current ED figures described in the literature [30] in similar populations [19, 26–28].

Our study results are in line with previous findings in other international settings on the validity and ease of use of 4AT in routine practice [21, 28]. Unlike many other instruments that evaluate the incidence and/or severity of delirium in different settings (palliative care [36], critical care units [37], residential homes, hospitalization units [38], ED [20, 39], etc.), the 4AT does not require any specific qualification or prior training. Another advantage is that it is a brief instrument that can be completed in less than 2 min. This factor is essential to the success of strategies for the detection and management of delirium in EDs, given the usual pressure of care and insufficient time to carry out exhaustive evaluations. Another added value is that emergency nurses can easily use it without dedicating too much time to the process, facilitating longitudinal assessments during the patient’s stay in the ED and monitoring more accurately the possible incidence of delirium.

In our study, emergency nurses with no prior training collected 4AT data. The validity results show that 4AT is easy to use in the ED and that it is immediately accessible to professionals such as nurses who have the most contact with patients. We minimized time differences between the administration of 4AT and the gold standard test to avoid any fluctuation in the patients’ condition, which is characteristic of delirium.

However, while an instrument such as 4AT can help detect delirium, pathways for the appropriate systematic management of this problem and prevention of adverse outcomes must also be implemented in the ED [40].

### Limitations

The ED, with its lack of privacy, noisy environment, and changes in patient location, was not conducive environment to performing evaluations, although is the real context where delirium assessment has to be developed. No assessments were performed during the night shift, which may have led to underdetection of delirium during these time periods.

In contrast to other studies [26], pre-existing versus incident delirium was not analyzed, and individuals with moderate-severe cognitive impairment, dementia or severe mental disorder were excluded to avoid sensitivity bias [27]. This implies a loss of external validity, since our findings cannot be extended to patients in the ED who present these conditions.

## Conclusion

The 4AT in Spanish is accurate and reliable for detecting delirium in elderly patients in the ED, and can be administered very easily by different healthcare professionals, including emergency nurses. The results of this study support the routine use of the 4AT score to detect delirium in the ED. This instrument addresses the difficulty to assess delirium in a time-scarce setting such ED since is an easy, quick and reliable method to evaluate this important challenge for ED nurses. Early detection of delirium helps to decrease length of stay, in-hospital mortality, disabilities, dementia development, and falls.

The 4AT Spanish version helps to detect delirium from the moment of ER admission, enabling appropriate preventive measures to be activated, both immediately and throughout the hospital stay.

Finally, it will help to increase awareness among healthcare professionals regarding the importance of early detection of this event, and may serve as a basis for future experimental studies to evaluate multicomponent interventions aimed at reducing the incidence of delirium, and at fostering a different approach towards healthcare for patients at the moment of admission.

## Data Availability

The datasets used and/or analyzed during the current study are available from the corresponding author on reasonable request.
